# Self-consistent recurrent neural network for path dependent deformation

**DOI:** 10.1038/s41598-026-49661-2

**Published:** 2026-05-02

**Authors:** Muhammed Adil Yatkin, Mihkel Kõrgesaar, Vedat Mert Asan, Jani Romanoff, Joshua Stuckner, Hasan Kurban

**Affiliations:** 1 School of Engineering, Kuressaare College, Tallinn University of Technology, Tallinn, Estonia; 2 Mechanical Engineering, Aalto University, Espoo, Finland; 3 NASA Glenn Research Center, Cleveland, USA; 4 College of Science and Engineering, Hamad Bin Khalifa University, Doha, Qatar

**Keywords:** Deep learning, Recurrent neural networks, Damage initiation in sheet metal, Fracture modelling, Non-proportional loading, Surrogate models, Engineering, Mathematics and computing

## Abstract

Using data-driven machine learning (ML) models as surrogates in classical engineering is an emerging trend in the literature. However, effective surrogate modeling in path-dependent problems requires a deep understanding of the fundamental physical properties that naturally arise in data obtained from simulations or experiments. While generic ML architectures can capture nonlinear behavior, they may not inherently satisfy the specific temporal constraints dictated by physical processes. This study examines the characteristics of deformation paths generated through finite element simulations and identifies key modeling requirements for achieving physically meaningful predictions. One important requirement is that future inputs do not influence past outputs, a property typically satisfied by most surrogate ML models, yet rarely acknowledged or formalized. This requirement, often called the truncation condition, is essential for achieving physically meaningful predictions. Another closely related requirement is consistency across different time discretizations, which remains an active and important topic in deformation history modelling. To address these requirements, we propose a customized and adaptable Recurrent Neural Network (RNN) transition function that takes absolute strain inputs and is designed to enforce both truncation and consistency, ensuring robust predictions across varying temporal resolutions. This study contributes toward improving physically consistent damage initiation estimation and supports the development of more reliable surrogate models in computational mechanics.

## Introduction

### Finite element (FE) methods and machine learning (ML) based surrogate modeling

Finite element (FE) simulations are widely used in engineering for studying the mechanical behavior of structures. When this mechanical behavior includes large deformations, plasticity, and fracture, simulations must account for the path-dependent nature of plastic materials^1^. For path-dependent material plasticity, the current state at a material point uniquely depends on the constitutive history, that is, the sequence of stress–strain increments experienced throughout the entire deformation process.

Given that the constitutive model describing the stress–strain response of the material is known (with all its microstructural features) and uniquely defined across the continuum, the solution to the continuum boundary value problem is also uniquely determined. While this setting can be achieved with micromechanical or unit-cell models (e.g., Ref.^2^), extending the principle to structural-scale engineering simulations poses challenges^3^. In practice, structural analyses rely on shell elements with grid spacing (FE element length) that are too large to resolve all microstructural details, and instead represent the material through an averaged macroscopic response.

To capture microstructural effects in shell response, multiscale methods could be used to achieve high accuracy^4^, but at a significant computational cost, making them impractical for routine engineering use^5^. In a coupled multiscale framework, the coarse shell element model supplies boundary conditions (kinematic fields) for a parallel numerical simulation at a much finer scale to resolve the local (non-uniform, high-fidelity) material response and history-dependent damage state. These results are then used as prescriptions for the coarse-grid constitutive model to resolve macroscopic stress–strain pairs. This exemplifies the two-way coupling strategy of multiscale models.

Yatkin and Kõrgesaar^6^ showed that this computationally demanding multiscale approach can be replaced by a one-dimensional convolutional neural network (1D CNN) surrogate while maintaining high accuracy. The model captures deformation history and its effect on the material damage state. The approach relies on three assumptions: (1) the structural model resolves macroscopic strains correctly, (2) the surrogate ML model delivers computational efficiency, and (3) the ML model can be embedded in an explicit FE code.

As the problem itself is inherently sequential, RNNs are well suited for learning mappings between input and output sequences, such as the stress–strain relation that reflects deformation history. For example, Mozaffar et al.^7^ showed that RNNs can simplify complex formulations arising from plasticity theory, while Wu et al.^8^ demonstrated that RNNs can act as surrogates for mesoscale boundary value problems (BVPs) in multiscale modeling. Beex et al.^9^ showed that a two-layer Gated Recurrent Unit (GRU) network can represent elastoplastic constitutive behavior under path-dependent deformation. Other studies have explored related architectures. Patra et al.^10^ applied several Long Short-Term Memory (LSTM) based models to predict heterogeneous deformation in dual-phase microstructures. Mao et al.^11^ proposed Convolutional Long Short-Term Memory (ConvLSTM) networks as surrogates to accelerate phase-field simulations. Huang et al.^12^ demonstrated that surrogate models trained on fine-scale simulations can be successfully integrated back into macro-scale FE environments. Ghavamian and Simone^13^ also used RNNs to accelerate multiscale FE simulations involving history-dependent materials by replacing micromechanical evaluations. Similarly, deep neural networks trained on FE data have been applied to predict microstructural evolution and optimize hot forging parameters, showing how simulation-informed surrogates can improve both accuracy and efficiency^14^. More broadly, several studies have enriched or replaced conventional constitutive models with data-driven approaches^15–22^.

More complex architectures have also been proposed. Tabarraei et al.^23^ employed a mixed neural network (NN) architecture combining 2D convolutional layers (2D CNNs), bidirectional RNNs, and fully connected layers to predict crack growth in close agreement with molecular dynamics simulations. Alahyarizadeh et al.^24^ developed a U-Net-based NN architecture^25^ as a surrogate model to reduce computational costs associated with phase-field-based simulations of microstructure evolution.

Despite these developments, damage estimation based on learned deformation history remains relatively unexplored. More importantly, the fundamental requirements that NN models must satisfy for deformation history based modeling have not been clearly addressed. In this study, we identify these essential requirements and propose a Customized Recurrent Neural Network ( C-RNN ) formulation for predicting the material damage state from deformation history.

**Fig. 1:**
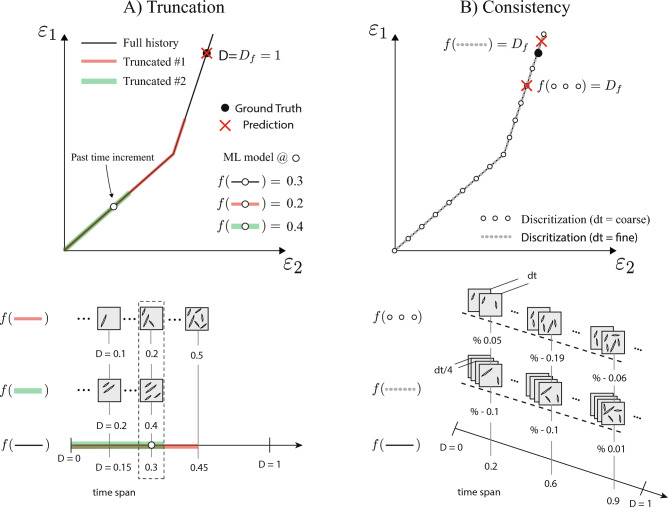
Illustration of the two essential requirements for physically consistent surrogate models. (**A**) Truncation : The diagram shows that when the ML model is fed with different history sequences of inputs from past data, it yields different damage predictions for the same deformation state, which violates the physical property of the data. (**B**) Consistency: The illustration shows that when the ML model is fed with different time frequencies for the same input path, it can give different damage predictions for the same deformation state, which also violates the fundamental requirement.

These essential requirements **truncation** and **consistency** ensure that the estimates of the NN architecture remain compatible with the intrinsic physical properties of deformation paths. One of these physical properties is the uniqueness of damage predictions: ongoing deformation should not affect damage state predictions already made by the NN in the past. This is crucial because we presuppose that damage is path dependent the current state is influenced by all deformation states that have already taken place.

Compliance with the **truncation** requirement ensures that damage predictions for each historical state are unique. Violation of this requirement is illustrated in Fig. 1A, where three different states of the same path are shown. The same mechanical state (marked with a black marker) is predicted using only a partial deformation history. The resulting predictions vary depending on how much of the history is provided, highlighting the sensitivity to truncation. Therefore, truncation is critical during both the training and inference phases of an NN model. While many surrogate models do not conflict with this requirement, few explicitly identify or discuss it. The uniqueness problem can arise, for instance, when encoder–decoder–based NN architectures are used as surrogates, since their latent representations depend on the entire input sequence.

The goal of the **consistency** requirement is to ensure that, while the NN model is trained on data with one discretization level (e.g., larger time increments), it still provides consistent predictions when applied to data with smaller increments during inference. In other words, the surrogate NN must remain increment-size independent. This concept was first postulated for elasto-plastic solids by Bonatti and Mohr^26^. Because of the incremental nature of numerical simulations, the number of increments can vary depending on the loading and boundary conditions. For a surrogate model to be applicable in such settings, its damage predictions must not depend on the chosen increment size. If the NN architecture is not designed to yield consistent predictions, physically incompatible results may arise, as shown in Fig. 1B, where varying discretizations of the same input path yield different damage predictions. Ensuring consistency is thus essential for physically reliable surrogate modeling.

Therefore, the contributions of this study can be summarized as follows :

- Two fundamental requirements, **truncation**, and **consistency**, are defined for an ML surrogate model to be compatible with the physical properties of deformation paths. Although there are studies that develop suitable surrogate models, to the best of our knowledge this is the first work that explicitly defines these two fundamental requirements.
- A customized Recurrent Neural Network (RNN) formulation is introduced to satisfy these requirements and provide accurate damage predictions when used as a surrogate ML model.
- The proposed approach is tested on both simulated and synthetic datasets, demonstrating that the method works reliably and can potentially be applied to other fields as well.
- The conclusions, limitations of the current approach, and future research directions are discussed to guide further development of physically consistent surrogate models.

This study is structured as follows. Section Method outlines the background, and previous work, Section Experiments presents the method, and proposed tailored RNN formula, and its heuristic-based theoretical construction. Section Conclusion reports validation experiments conducted on both synthetic and physics-based simulation dataset, followed by a discussion of the results. Section 5 concludes the study and outlines directions for future work.

**Fig. 2:**
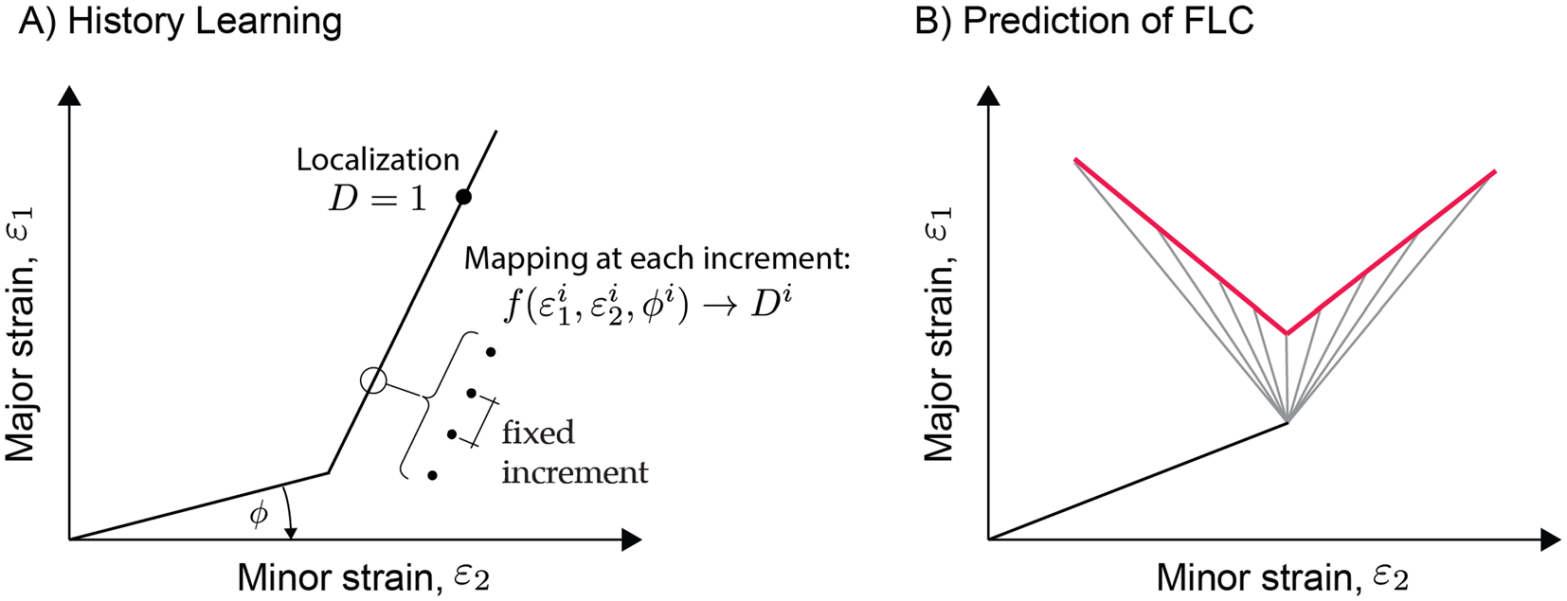
The mapping defined in Yatkin and Kõrgesaar^6^ from bilinear strain paths to the damage indicator *D*..

### Background and prior work

In our earlier work^6^, a bilinear loading dataset was generated using physics-based Marciniak–Kuczynski (MK) FE simulations. The dataset comprised 19,098 bilinear, non-proportional loading paths, each consisting of two proportional segments with known characteristics Fig. 2A. Each path contained 400 increments sampled at a fixed increment size.

The equivalent plastic strain $\bar{\varepsilon }_{i}$ was computed at each increment as

1 $$\begin{aligned} \bar{\varepsilon }_{i}=\int _0^i \textrm{d}\bar{\varepsilon }, \quad \text {where} \quad \textrm{d}\bar{\varepsilon }=\frac{2}{\sqrt{3}}\sqrt{(\textrm{d}\varepsilon _1)^2+(\textrm{d}\varepsilon _{2})^2+(\textrm{d}\varepsilon _1)(\textrm{d}\varepsilon _{2})}, \end{aligned}$$

Here, $\textrm{d}\varepsilon _1$ and $\textrm{d}\varepsilon _2$ represent the incremental strain differences in the major and minor strain directions, respectively, computed between two consecutive time steps along the bilinear loading paths, and the corresponding damage indicator $D^{i}$ was evaluated as

2 $$\begin{aligned} D^{i} =\frac{\bar{\varepsilon }_i}{\bar{\varepsilon }_{\text {fail}}}, \quad i \in \{1,2,\dots ,400\}. \end{aligned}$$

The sequential ML formulation mapped the strain state and loading direction $(\varepsilon _1^i, \varepsilon _2^i, \phi ^i)$ to a damage indicator $D^i$ using a 1D CNN based NN architecture trained on deformation histories. The trained model was subsequently employed to predict three different forming limit curves (FLCs) Fig. 2B, shaped by 120 distinct bilinear loading paths.

In contrast to the 1D CNN based model used in our previous study^6^, recent research^10,27–29^ on material modeling has increasingly preferred other deep learning–based approaches as surrogates for constitutive behavior to capture deformation history in numerical simulations. While 1D CNN based architectures effectively capture local patterns in sequential data, RNNs are better suited for learning temporal and long-term dependencies which is more relevant for history learning related to load paths in mechanical engineering.

FE–based numerical simulations rely on incremental processing, conceptually similar to how RNNs process sequential data step-by-step. Conversely, 1D CNNs operate on entire sequences simultaneously, applying convolutional filters to the full input to extract local patterns via receptive fields. This simultaneous processing limits their ability to handle time-dependent, stepwise data, since they lack a mechanism to maintain and update internal states over time.

To address the limitations of the 1D CNN based surrogate model, this study introduces a C-RNN formulation designed to align with the physical characteristics of deformation paths. The proposed RNN architecture is evaluated on both synthetic and physics based simulation derived datasets. Specifically, the model is trained on bilinear deformation path data, and validated using three forming limit curve (FLC) cases simulated with finer temporal resolutions than in our previous study^6^. The performance and consistency of the model are subsequently analyzed and discussed, followed by an outline of potential directions for future research.

## Method

In the current study, the damage evolution defined as a mapping in strain space, where the evolution of the damage variable is governed by the relation as

3 $$\begin{aligned} f: (\varepsilon _{1}^{t}, \varepsilon _{2}^{t}, \phi ^{t}) \mapsto D^{t} \end{aligned}$$

Here, *f* represents a Customized RNN-based NN architecture, and the hidden state of the RNN represents a history-dependent internal material state that retains the path-dependent loading information accumulated during deformation. It is updated incrementally based on the major and minor strain inputs, and the direction of the loading at each time step. Therefore, during each incremental update, the RNN operates similarly to an internal material evolution law in classical constitutive modeling, where the memory of the loading path is retained and the damage variable is obtained through a state transition mechanism.

To ensure predictions consistent with the physical state of deformation history, NN architectures can be designed to embed implicit physical constraints. Two key requirements are identified: (1) the **truncation** requirement, meaning that predictions generated for past increments must remain unaffected by future inputs, and (2) the **consistency** requirement, meaning that damage estimates for a given loading path should be independent of the discretization size used to represent it. In numerical simulations, the number of increments for a given path can vary depending on the time-step size. These two requirements form the foundation for defining the conditions an NN architecture must satisfy to produce physically meaningful predictions.

Since the defined fundamental requirements, namely truncation and consistency, need to be satisfied by any sequential learning method such as Transformers and 1D-CNNs to be used as a surrogate inside FE simulations, here we explore these requirements within Recurrent Neural Networks (RNNs). Due to their incremental learning and prediction structure, RNNs are compatible with the mathematical calculations that take place inside finite element solvers, as also stated in Mohr’s research works^26,30^. Therefore, the state update mechanism of RNNs is well suited to represent the evolution of material states and to capture the memory of the deformation path.

In the previous study^31^, we proposed a specialized RNN formulation designed to provide consistent estimates using incremental inputs of the deformation paths, inspired by the consistency framework proposed by Bonatti and Mohr^26^. That earlier approach relied on incremental strain differences, similar to their formulation. In contrast, the present study introduces a C-RNN formula that directly accepts *absolute strain values* at each time step rather than their incremental difference. By embedding both **truncation** and **consistency** requirements into the RNN architecture, the proposed method serves as a robust surrogate model capable of accurately predicting the initiation of damage for bilinear forming limit curves (FLCs).

### Truncation

The first requirement, shown in Fig. 1A, is formulated as follows. Given the inputs $(x_{1}, x_{2}, \dots , x_{N})$ to the NN architecture, the corresponding predicted outputs are $(y_{1}, y_{2}, \dots , y_{N})$. A new input $x_{N+1}$ should not affect any of the previously predicted outputs. Formally, this can be expressed as:

4 $$\begin{aligned} \begin{aligned} (x_{1}, x_{2}, \dots , x_{N})&\rightarrow NN \rightarrow (y_{1}, y_{2}, \dots , y_{N}), \\ (x_{1}, x_{2}, \dots , x_{N}, x_{N+1})&\rightarrow NN \rightarrow (y_{1}, y_{2}, \dots , y_{N}, y_{N+1}). \end{aligned} \end{aligned}$$

Therefore, when a new input point $x_{N+1}$ is provided, the model should reproduce all previous outputs $y_i$ unchanged.

Violation of this property can be expressed as:

5 $$\begin{aligned} \begin{aligned} (x_{1}, x_{2}, \dots , x_{N})&\rightarrow NN \rightarrow (y_{1}, y_{2}, \dots , y_{N}), \\ (x_{1}, x_{2}, \dots , x_{N}, x_{N+1})&\rightarrow NN \rightarrow (\hat{y}_{1}, \hat{y}_{2}, \dots , \hat{y}_{N}, \hat{y}_{N+1}), \quad \text {where } y_{i} \ne \hat{y}_{i} \text { for } i \le N. \end{aligned} \end{aligned}$$

Violating the **truncation** requirement can lead to different predicted damage initiations for identical deformation states, as shown in Eq. (5). This behavior is common in encoder–decoder based RNNs, whose hidden states are globally dependent on all input history points, and thus violate **truncation** requirement.^32^.

### Consistency

If an NN surrogate model is fed with a different number of increments for the same deformation path, the estimated damage initiation points should remain consistent at the same physical location. However, as Bonatti and Mohr^26^ noted, the exact **consistency** is not possible, since the NN computations are inherently statistical, relying on probability distributions represented by the learned weights after training. Therefore, consistency in this context must be interpreted as approximate **consistency**. Based on this reasoning, the requirement can be expressed mathematically as Eq. (6).

6 $$\begin{aligned} \begin{aligned} \text {For } M, N, i, j \in \mathbb {Z}, \ \text {such that }&M > N \text { and } i \ne j, \\ \text {if } X = (x_{1}, x_{2}, \dots , x_{N})&\rightarrow NN \rightarrow Y = (y_{1}, y_{2}, \dots , y_{N}); \ y_{i} = 1, \\ \text {and } X' = (x_{1}, x_{2}, \dots , x_{M})&\rightarrow NN \rightarrow Y' = (y'_{1}, y'_{2}, \dots , y'_{M}); \ y'_{j} = 1, \\ \text {then }&\frac{i}{N} \approx \frac{j}{M}. \end{aligned} \end{aligned}$$

In Eq. (6), *X* and $X'$ represent different incremental discretizations of the same deformation path. Although the same path may be represented with different numbers of increments (*M* and *N*), the predicted point of damage initiation ($y_{i}=1$, $y'_{j}=1$) should correspond approximately to the same physical location along that path.

**Fig. 3:**
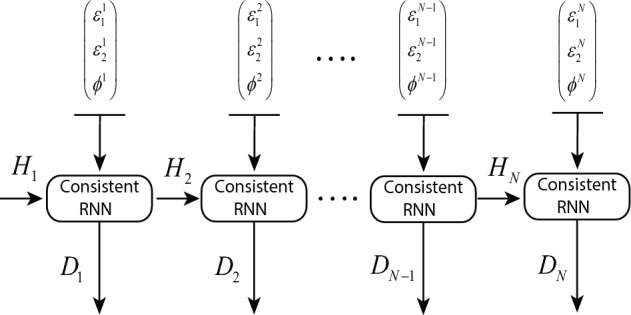
Sequential neural network architecture built from RNN layers containing consistent cells. As shown in our previous study^32^, encoder–decoder RNNs exhibit truncation issues: with truncated input histories, the decoder initializes from a different hidden state, leading to variations in predicted damage initiation. By contrast, the proposed architecture eliminates this dependency and provides both **truncation** and **consistency**.

To ensure predictions that are compatible with the physical state of deformation even under truncated history input we structured the NN architecture as shown in Fig. 3, using a specialized RNN layer. In this structure, each estimate is computed from a unique hidden state that depends solely on the previous history. Thus, hidden states associated with earlier inputs are not affected by future time-step data. This causal computation eliminates the **truncation** issue illustrated in Fig. 1A and ensures that estimates remain consistent with the physical properties of deformation paths.

#### Consistency through RNNs with a customized transition function

While the consistency problem has been discussed in the work of Bonatti and Mohr^26^, their proposed solution was specifically tailored for constitutive modeling applications, where the RNN inputs must be expressed as incremental differences in strain space. In contrast, we propose a heuristic approach that can be adapted to any sequential modeling framework where the inputs are absolute time-step values rather than incremental differences.

RNNs operate by updating a hidden state vector at each increment during training, encoding the temporal evolution of the input sequence. This process is governed by the RNN’s transition function, which determines how the state evolves from $H_{t-1}$ to $H_t$. To achieve consistency between predictions from differently discretized inputs representing the same strain path, a customized transition function must be designed.

#### Theoretical hypothesis

**Fig. 4:**
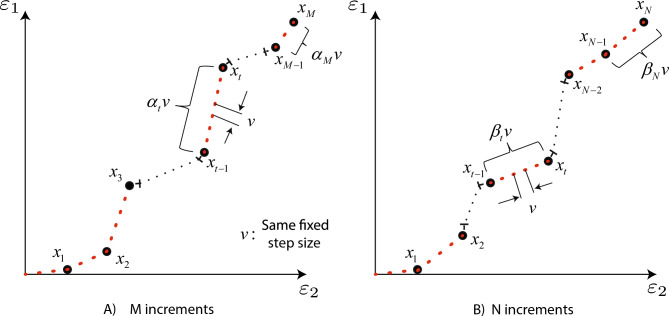
Different numbers of increments *M*, and *N* representing the same multilinear loading path in strain space.

A continuous path in strain space, sampled with two different increment sizes, can be represented by two sets of points containing *M* and *N* increments, respectively Fig. 4A and B. If the path between any two arbitrary increments is assumed to be linear, there exists a sufficiently small step size *v* that can divide all incremental differences into positive integer numbers of smaller steps. The integers $\alpha _i$ and $\beta _j$ ($i \in \{1,\dots ,M\}$, $j \in \{1,\dots ,N\}$) represent the number of small steps required to complete each linearized segment of the path.

The total strain increment accumulated along each discretized representation must be approximately equal, as expressed in Eq. (7).

7 $$\begin{aligned} v\alpha _{1} + v\alpha _{2} + \dots + v\alpha _{M} \approxeq v\beta _{1} + v\beta _{2} + \dots + v\beta _{N} \end{aligned}$$

**Fig. 5:**
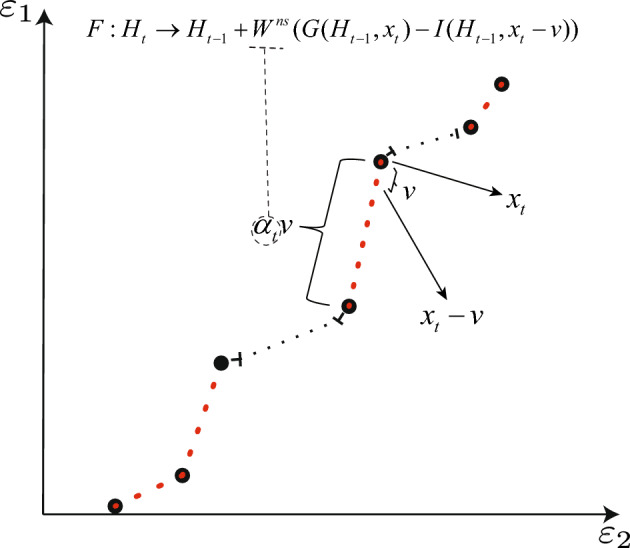
Theoretical intuition behind the customized transition function. Here, $G(H_{t-1}, x_t) - I(H_{t-1}, x_t - v)$ captures the small-step update *v*, while $W^{ns}$ learns the number of steps needed to complete each increment.

To ensure consistent estimates from the RNN transition function, we formulated a modified update rule, Eq. (8), based on the theoretical intuition illustrated in Fig. 5. Here, *G* and *I* are structurally similar transition functions with distinct weight matrices that learn how small variations in input affect the hidden state. The difference $G(H_{t-1}, x_t) - I(H_{t-1}, x_t - v)$ learns the local update associated with a small step *v*, while the weight matrix $W^{ns}$ approximates how many such small steps occur between time steps $t-1$ and *t*.

8 $$\begin{aligned} F(H_{t-1}, x_{t}): H_{t} \rightarrow H_{t-1} + W^{ns}\big (G(H_{t-1}, x_{t}) - I(H_{t-1}, x_{t} - v)\big ) \end{aligned}$$

The heuristic concept is to linearize each path segment in the loading direction and to learn this linearized evolution via small, consistent updates. The transition formula in Eq. (8) therefore allows the model to estimate how many micro-steps are required to move from one state to the next, and how each small step modifies the state vector. Multiplying these two approximations and adding them to the previous state produces a consistent total update.

#### Examination of consistency based on trajectories in state space

Because the RNN approximates the evolution between successive data points, exact consistency is unrealistic. However, by emulating the mathematical structure of perfect consistency, an approximately consistent transition rule can be achieved. The primary goal is to ensure that the model trained on coarse increments provides accurate predictions when evaluated with finer discretizations.

**Fig. 6:**
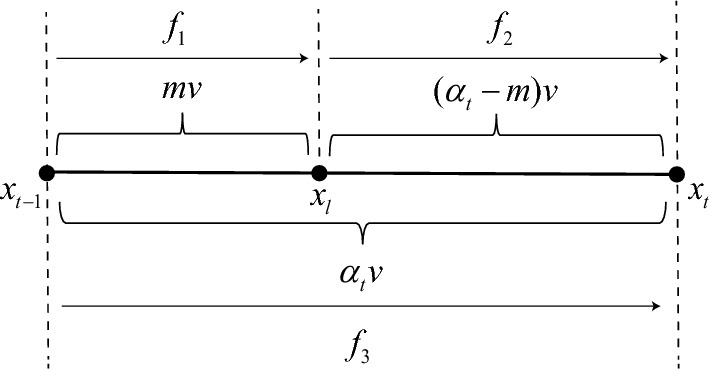
Incremental update representation at time step *t*. Here, $\alpha _t$ denotes the number of micro-steps and *v* the step size. For consistency, the RNN transition function must satisfy Eq. (9) when using intermediate inputs $x_l$ between $x_{t-1}$ and $x_t$.

During the incremental update from time step $t-1$ to *t* (Fig. 6), if the state $H_{t-1}$ is first updated with an intermediate input $x_{l}$ (between $x_{t-1}$ and $x_t$) and then updated with $x_t$, the resulting $H_t$ should match the state obtained by directly updating $H_{t-1}$ with $x_t$. This is expressed in Eq. (9). The proposed transition function in Eq. (8) satisfies this condition under the assumption in Eq. (10), where each small step has approximately the same effect on the hidden state between two successive inputs.

9 $$\begin{aligned} F(F(H_{t-1}, x_l), x_t) = F(H_{t-1}, x_t) \end{aligned}$$

10 $$\begin{aligned} G(H_{t-1}, x_l) - I(H_{t-1}, x_l - v) \approxeq G(H_l, x_t) - I(H_l, x_t - v) = \Theta \end{aligned}$$

To demonstrate that the transition function *F* in Eq. (8) ensures consistency, we define three trajectories for the incremental update from time step $t-1$ to *t*, as shown in Eq. (11). These trajectories correspond to linear micro-step updates with counts *m* and $\alpha _t$ (Fig. 6). Specifically, $f_1$ represents the update from $x_{t-1}$ to an intermediate input $x_l$, $f_2$ the update from $x_l$ to $x_t$, and $f_3$ the direct update from $x_{t-1}$ to $x_t$. Note that the initial state vector $H_{t-1}$ is shared by $f_1$ and $f_3$.

11 $$\begin{aligned} \begin{aligned}&f_1:\; F(H_{t-1}, x_l) = H_{t-1} + W^{ns}\!\big (G(H_{t-1}, x_l) - I(H_{t-1}, x_l - v)\big ) \approx H_{t-1} + m\Theta = H_l, \\&f_2:\; F(H_l, x_t) = H_l + W^{ns}\!\big (G(H_l, x_t) - I(H_l, x_t - v)\big ) \approx H_l + (\alpha _t - m)\Theta , \\&f_3:\; F(H_{t-1}, x_t) = H_{t-1} + W^{ns}\!\big (G(H_{t-1}, x_t) - I(H_{t-1}, x_t - v)\big ) \approx H_{t-1} + \alpha _t\Theta . \\ \end{aligned} \end{aligned}$$

12 $$\begin{aligned} f_2 \;\approxeq \; f_3. \end{aligned}$$

Applying the assumption in Eq. (10) to the trajectories in Eq. (11) shows that $f_2$ and $f_3$ are approximately equal, as stated in Eq. (12). Hence, the consistency condition implied by Eq. (8) holds, and the transition function *F* in Eq. (8) is approximately consistent.

## Experiments

The central hypothesis behind the RNN transition formula in Eq. (8) suggests a possible route toward achieving consistency. It is therefore essential to test the C-RNN cell on datasets with diverse strain paths. Since the bilinear loading dataset covers only specific loading scenarios in strain space, we additionally generated a synthetic dataset with broader representational coverage to compare standard GRU- and LSTM-based NN architectures with the proposed C-RNN cell-based NN architecture.

We implement the transition formula in Eq. (8) separately for the synthetic and bilinear datasets. The synthetic implementation serves as a proof of concept using basic functions, whereas the bilinear implementation is more specialized.

### Synthetic dataset

The proposed consistency hypothesis may extend to datasets characterized by continuous path relationships. To examine this, Eq. (8) is first evaluated using a synthetically generated dataset that provides broader coverage than the bilinear loading example.

#### Synthetic path generation

**Fig. 7:**
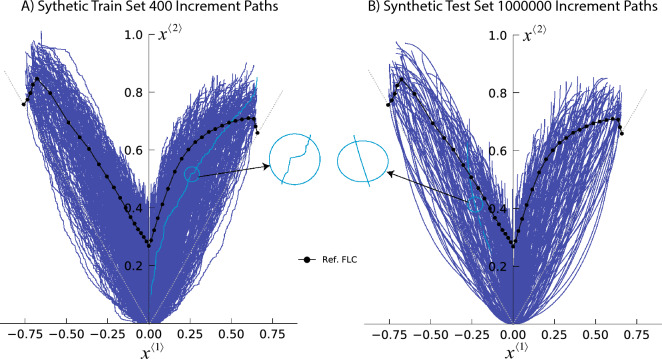
(**A**) Random multilinear paths (400 increments) with endpoints just beyond the Reference FLC, used for training the specialized RNN cell. (**B**) Additional randomly generated paths with 1, 000, 000 increments for quantitative consistency assessment.

To generate synthetic paths, we take the endpoints of the first loading segments from the bilinear dataset of Yatkin and Kõrgesaar^6^ (used to define the Reference FLC). We then randomly generate 35, 711 multilinear paths $P_s$ of length 400 increments with endpoints immediately beyond the Reference FLC (Fig. 7A). For each path, the synthetic equivalent plastic strain $\varepsilon _s^q$ and the synthetic damage indicator $D_s$ are computed using the intersection index with the Reference FLC (Eqs. (14)–(15)), yielding the mapping in Eq. (16). The dataset is split into $75\%$ training, $15\%$ test, and $10\%$ validation. To quantitatively assess consistency, we also generate 400 multilinear paths with 1, 000, 000 increments using the same randomization (Fig. 7B).

13 $$\begin{aligned} \begin{aligned} P_{s}&: \text {randomly generated multilinear path}, \\ P_{s}&= \big [(x_{1}^{\langle 1 \rangle }, x_{1}^{\langle 2 \rangle }), (x_{2}^{\langle 1 \rangle }, x_{2}^{\langle 2 \rangle }), \dots , (x_{400}^{\langle 1 \rangle }, x_{400}^{\langle 2 \rangle })\big ], \quad s \in \{1,\dots , 35711\}, \\ (\Delta a_i)_s&= x_{i}^{\langle 1 \rangle } - x_{i-1}^{\langle 1 \rangle }, \quad (\Delta b_i)_s = x_{i}^{\langle 2 \rangle } - x_{i-1}^{\langle 2 \rangle }, \quad i \in \{1,\dots ,400\}. \end{aligned} \end{aligned}$$

14 $$\begin{aligned} \varepsilon ^{q}_{s} = \sum _{j=1}^{q} \frac{2}{\sqrt{3}}\!\left( (\Delta a_{j})_{s}^{2} + (\Delta b_{j})_{s}^{2} + (\Delta a_{j})_{s}(\Delta b_{j})_{s}\right) , \quad q \in \{1,\dots ,400\}. \end{aligned}$$

15 $$\begin{aligned} \begin{aligned} k&\;=\; \text {intersection index with Reference FLC}, \\ D_{s}&= \big [\varepsilon ^{1}_{s}/\varepsilon ^{k}_{s},\; \varepsilon ^{2}_{s}/\varepsilon ^{k}_{s},\; \dots ,\; \varepsilon ^{400}_{s}/\varepsilon ^{k}_{s}\big ]. \end{aligned} \end{aligned}$$

16 $$\begin{aligned} P_{s} \;\longrightarrow \; D_{s}. \end{aligned}$$

#### Implementation details for the synthetic dataset

Because *G* and *I* in Eq. (8) can be chosen freely, we adopt a basic RNN transition for the synthetic dataset (Eq. (17); cf.^33^). Given that the synthetic paths lack specific structural patterns, a simple RNN suffices. We use $\tanh$ for the state activation (Eq. (18)), and compute the output through a linear layer followed by a sigmoid (Eq. (19)).

17 $$\begin{aligned} \begin{aligned} G(H_{t-1}, x_t)&= \sigma \!\left( W^{gx}x_t + W^{gg}H_{t-1} + W_{0}^{gg}\right) , \\ I(H_{t-1}, x_t - v)&= \sigma \!\left( W^{hx}(x_t - v) + W^{hh}H_{t-1} + W_{0}^{hh}\right) , \end{aligned} \end{aligned}$$

18 $$\begin{aligned} F(H_{t-1}, x_t) \;=\; \tanh \!\Big (H_{t-1} + W^{ns}\!\big (G(H_{t-1}, x_t) - I(H_{t-1}, x_t - v)\big )\Big ). \end{aligned}$$

19 $$\begin{aligned} \begin{aligned} H_t&= F(H_{t-1}, x_t), \\ y_t&= \sigma \!\left( W^{oo}H_t + W_{0}^{oo}\right) . \end{aligned} \end{aligned}$$

Since the objective is to learn the mapping in Eq. (16), the input to the specialized RNN cell at time *t* for a given synthetic path $P_s$ is

20 $$\begin{aligned} \begin{aligned} x_t&= \big (x_{t}^{\langle 1 \rangle },\; x_{t}^{\langle 2 \rangle }\big ), \\ x_t - v&= \big (x_{t}^{\langle 1 \rangle } - v,\; x_{t}^{\langle 2 \rangle } - v\big ). \end{aligned} \end{aligned}$$

#### Training on Synthetic Dataset

A single-layer *GRU*-based and *LSTM*-based NN architectures were constructed and hyper-tuned over 20 trials, varying batch size, learning rate, hidden-state size, and optimizer. The output layer is a fully connected layer. Batch sizes {32, 64, 128} were tested; learning rates were explored in $[10^{-2},\,10^{-4}]$; and hidden units in {32, 64, 128, 192, 256}. Optimizers included Adam, RMSprop, and SGD. Bayesian optimization was used to identify the best hyperparameter combination for convergence. Detailed results are shown in Tables 1 and  2.

**Table 1:** Comprehensive hyperparameter trials for the GRU model across the Bilinear and Synthetic datasets.

Model	H	Opt.	LR	Trial	Val MSE
Bilinear Dataset					
GRU	128	adam	0.001111	9	**0.000542**
GRU	192	rmsprop	0.001492	8	0.000548
GRU	256	adam	0.002191	10	0.000562
GRU	32	adam	0.006428	12	0.000565
GRU	32	adam	0.000924	13	0.000665
GRU	256	adam	0.0004399	18	0.000691
GRU	256	adam	0.0001	15	0.000706
GRU	96	adam	0.0001	16	0.000733
GRU	160	adam	0.01	14	0.000742
GRU	32	adam	0.0001765	5	0.000808
GRU	192	adam	0.0001427	11	0.000856
GRU	32	adam	0.0001	19	0.001013
GRU	32	sgd	0.003129	7	0.011466
GRU	96	sgd	0.005663	3	0.013164
GRU	160	sgd	0.005181	6	0.013698
GRU	192	sgd	0.001546	4	0.014026
GRU	128	sgd	0.0009538	2	0.015246
GRU	256	rmsprop	0.01	17	0.018137
GRU	160	sgd	0.0006555	–	0.019098
GRU	64	sgd	0.0001408	1	0.038826
Synthetic Dataset					
GRU	192	adam	0.001803	12	**0.000646**
GRU	256	adam	0.01	10	0.000706
GRU	32	adam	0.01	17	0.000723
GRU	256	adam	0.0003945	9	0.000802
GRU	32	adam	0.0007269	8	0.000817
GRU	160	adam	0.0001	11	0.000837
GRU	64	adam	0.004425	4	0.000845
GRU	64	rmsprop	0.003456	3	0.000855
GRU	128	rmsprop	0.002614	5	0.000878
GRU	160	adam	0.01	16	0.000891
GRU	256	adam	0.0001	14	0.000946
GRU	32	adam	0.0001879	18	0.000968
GRU	128	rmsprop	0.0006787	1	0.000981
GRU	32	adam	0.0001	15	0.000999
GRU	32	adam	0.0001	19	0.001404
GRU	256	rmsprop	0.0001	13	0.002421
GRU	32	sgd	0.00907	6	0.008216
GRU	192	sgd	0.001609	2	0.012273
GRU	32	sgd	0.0001261	7	0.028103

Each configuration reports validation mean squared error (MSE) under varying hidden sizes, optimizers, and learning rates. Trials are indexed for reproducibility. Lower values indicate better validation performance; best-performing configurations within each dataset are highlighted in bold.

**Table 2:** Comprehensive hyperparameter trials for the LSTM model across the Bilinear and Synthetic datasets.

Model	H	Opt.	LR	Trial	Val MSE
Bilinear dataset					
LSTM	128	adam	0.001111	9	**0.000542**
LSTM	32	adam	0.00882347	3	0.000541708
LSTM	96	adam	0.008 24128	8	0.000552434
LSTM	224	adam	0.000702258	7	0.000573721
LSTM	256	rmsprop	0.00180980	1	0.000582532
LSTM	32	adam	0.00113622	13	0.000677122
LSTM	32	rmsprop	0.00555342	4	0.000700749
LSTM	128	adam	0.0001	14	0.000726700
LSTM	160	adam	0.0001	18	0.000734720
LSTM	160	adam	0.0001	19	0.000787032
LSTM	192	adam	0.0001	16	0.000787536
LSTM	256	adam	0.0001	12	0.000794320
LSTM	160	adam	0.0001	17	0.000827291
LSTM	256	adam	0.01	10	0.000871293
LSTM	192	adam	0.000216644	2	0.000946408
LSTM	32	adam	0.000198429	9	0.001223769
LSTM	128	sgd	0.00987376	0	0.002201644
LSTM	256	sgd	0.01	11	0.002326077
LSTM	32	sgd	0.01	15	0.003025696
LSTM	256	sgd	0.000158289	6	0.037096288
LSTM	128	sgd	0.000113307	5	0.039145716
Synthetic dataset					
LSTM	32	adam	0.0032384659	13	**0.000704**
LSTM	64	adam	0.0021530605	11	0.000715
LSTM	64	adam	0.0026638236	16	0.000765
LSTM	64	adam	0.0033851182	00	0.000772
LSTM	64	adam	0.0022108968	06	0.000839
LSTM	64	adam	0.0018203345	17	0.000848
LSTM	64	adam	0.0021681469	15	0.000868
LSTM	224	adam	0.0002046976	09	0.000967
LSTM	224	adam	0.0001772245	04	0.001027
LSTM	128	rmsprop	0.0001298333	08	0.001189
LSTM	224	adam	0.0001906983	19	0.001192
LSTM	32	adam	0.0001169701	05	0.001192
LSTM	128	rmsprop	0.0001445304	14	0.001301
LSTM	160	rmsprop	0.0002244890	18	0.001411
LSTM	160	rmsprop	0.0001887575	10	0.001675
LSTM	256	sgd	0.0075986992	12	0.003275
LSTM	256	sgd	0.0066956081	01	0.003642
LSTM	224	sgd	0.0036990629	02	0.007384
LSTM	32	sgd	0.0001172275	03	0.023875
LSTM	32	sgd	0.0001154562	07	0.027535

Each configuration reports validation mean squared error (MSE) under varying hidden sizes, optimizers, and learning rates. Trials are indexed for reproducibility. Lower values indicate better validation performance; best-performing configurations within each dataset are highlighted in bold.

Second, a one-layer C-RNN based NN architecture was built using the specialized transition formula in Eq. (8), followed by a fully connected layer with a sigmoid activation. Hyperparameters were tuned with batch sizes in {64, 128, 192} and learning rates in $[10^{-2},\,10^{-4}]$. Early stopping (patience $=5$) was employed. The fixed micro-step *v* in Eq. (8) was set to 0.0001. Training used Adam with mean squared error (MSE) loss. Bayesian optimization guided up to 100 training epochs per trial. Trial-wise results are in Table 3.

**Table 3:** Hyperparameter evaluation of the Customized RNN (C-RNN) across Bilinear and Synthetic datasets.

Model	H	Opt.	LR	Trial	Val MSE
Bilinear Dataset					
C-RNN	64	adam	0.001	2	**0.000594**
C-RNN	64	adam	0.0005	3	0.000598
C-RNN	128	adam	0.001	5	0.000617
C-RNN	64	adam	0.001	6	0.000620
C-RNN	64	adam	0.0005	7	0.000620
C-RNN	128	adam	0.0005	4	0.000625
C-RNN	192	adam	0.0005	1	0.000645
C-RNN	192	adam	0.001	–	0.000790
Synthetic Dataset					
C-RNN	192	adam	0.0001	5	**0.001061**
C-RNN	64	adam	0.001	6	0.001099
C-RNN	128	adam	0.0005	4	0.001159
C-RNN	64	adam	0.0001	1	0.001277
C-RNN	192	adam	0.0005	2	0.001339
C-RNN	128	adam	0.0001	7	0.001342
C-RNN	128	adam	0.0001	3	0.001688
C-RNN	192	adam	0.001	–	0.002021

Each configuration lists hidden layer size, optimizer, learning rate, and trial index. Validation MSE quantifies model generalization, with the lowest values indicating superior performance. Best results within each dataset are highlighted in bold.

**Fig. 8:**
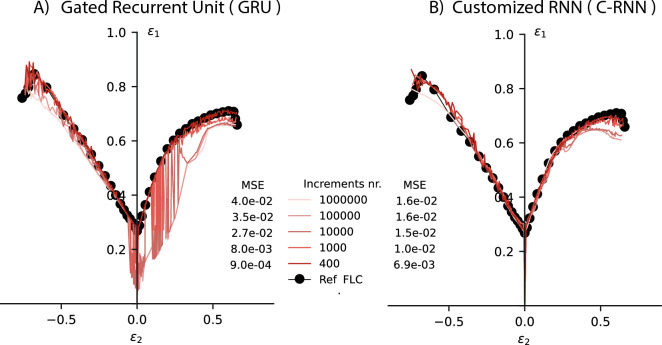
(**A**) GRU predictions diverge from the reference FLC as the number of increments increases. (**B**) Customized RNN predictions remain stable across changing discretizations, with a small accuracy drop.

The 1,000,000 increment paths shown in Fig. 7 were downsampled to 400, 1000, 10,000, and 100,000 increments using intervals of 2500, 1000, 100, and 10, respectively. As an overall trend, the GRU architecture exhibited performance similar to the LSTM models, while in several cases achieving slightly lower validation mean squared error values. Therefore, for comparison with the C-RNN, GRU-based predictions were used. Predictions from the best-performing GRU and Customized RNN-based NN architectures were obtained for each discretization. Using the index at which the predicted value first reaches 1, the corresponding coordinates were plotted in Fig. 8A and B.

#### Discussion of results

Figure 8A shows that GRU based NN architecture predictions diverging near boundary points as discretization increases, whereas the C-RNN based NN architecture estimations shown in Fig. 8 (B) maintains consistent across different discretizations. These results support the **consistency** hypothesis related to formula in Eq. 8.

A notable trade-off is that the C-RNN based NN architecture is less accurate for 400 increments than the GRU based architecture (Fig. 8B vs. A). While the proposed mechanism enforces discretization **consistency**, some accuracy is lost likely due to the absence of gating mechanism^34^ in Eq. (8). Although removing gates can raise concerns about vanishing gradients^35^, our objective learning a continuous path from few increments and extrapolating consistently to many increments mitigates this issue in practice.

### Bilinear loading dataset

Yatkin and Kõrgesaar^6^ generated bilinear FLCs (Fig. 2B) by rotating the second loading segment while fixing the first segment’s length and direction. FLCs indicate the maximal strain before localization, a proxy for ductile fracture initiation. In that work, both the bilinear paths and the FLC-generating paths used 400 increments. Here, we target consistent FLC estimates at *large* numbers of increments while training on *few*-increment bilinear paths.

We modify the synthetic-data cell for the bilinear setting and train an NN built from this cell on the 400-increment bilinear dataset. Additionally, three new bilinear FLC cases are simulated at a time frequency of 0.00001 Hz, producing 100, 000-increment bilinear paths.

#### Modified implementation details

For the bilinear case, instead of *G* and *I* from Eq. (17), we employ *Z* and *M* (Eqs. (21)–(22)), which mirror a standard GRU^36^:

21 $$\begin{aligned} \begin{aligned} Z(H_{t-1},x_{t})&= \sigma (W^{zx}x_{t} + W^{zz}H_{t-1} + W_{0}^{bz}), \\ R(H_{t-1},x_{t})&= \sigma (W^{rx}x_{t} + W^{rr}H_{t-1} + W_{0}^{br}), \\ \hat{S}(H_{t-1}, x_{t})&= \tanh \!\left( W^{sx}x_{t} + W^{ss}\big (R(H_{t-1},x_{t}) \otimes H_{t-1}\big ) + W_{0}^{bs}\right) , \\ G(H_{t-1},x_{t})&= (1 - Z(H_{t-1},x_{t})) \otimes H_{t-1} + Z(H_{t-1},x_{t}) \otimes \hat{S}(H_{t-1}, x_{t}). \end{aligned} \end{aligned}$$

22 $$\begin{aligned} \begin{aligned} M(H_{t-1},x_{t} - v)&= \sigma (W^{mx}(x_{t}-v) + W^{mm}H_{t-1} + W_{0}^{bm}), \\ N(H_{t-1},x_{t}-v)&= \sigma (W^{nx}(x_{t}-v) + W^{nn}H_{t-1} + W_{0}^{bn}), \\ \hat{K}(H_{t-1}, x_{t}-v)&= \tanh \!\left( W^{kx}(x_{t}-v) + W^{kk}\big (N(H_{t-1},x_{t}-v)\otimes H_{t-1}\big ) + W_{0}^{bk}\right) , \\ I(H_{t-1},x_{t}-v)&= (1 - M(H_{t-1},x_{t}-v))\otimes H_{t-1} + M(H_{t-1},x_{t}-v)\otimes \hat{K}(H_{t-1}, x_{t}-v). \end{aligned} \end{aligned}$$

**Fig. 9:**
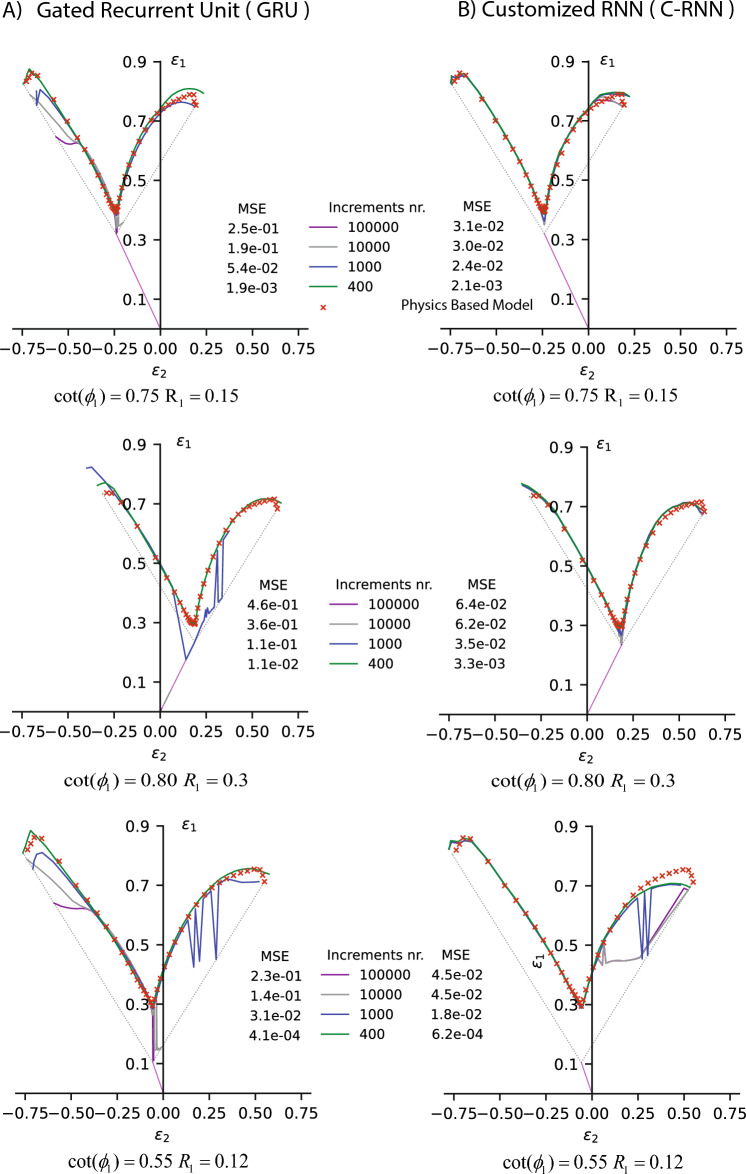
Bilinear Forming Limit Curve (FLC) estimates obtained from two NN architectures: (**A**) a GRU-based model and (**B**) the proposed C-RNN. The GRU predictions exhibit divergence as the number of increments increases, whereas the proposed RNN maintains consistent estimates across different strain-path discretizations.

Considering the mapping learned by the specialized RNN cell in strain space, as defined by Yatkin and Kõrgesaar^6^ (Fig. 2A), the input value at time step *t* for the specialized RNN is defined in Eq. (23). In each iteration, the hidden state is updated using a fixed step size *v* in the loading direction, multiplied by the estimated number of steps.

23 $$\begin{aligned} \begin{aligned} x_t&= (\varepsilon _{1,t},\, \varepsilon _{2,t},\, \phi _{t}), \\ x_t - v&= (\varepsilon _{1,t} - v\sin \phi _{t},\, \varepsilon _{2,t} - v\cos \phi _{t},\, \phi _{t}). \end{aligned} \end{aligned}$$

#### Training on bilinear loading dataset

One-layer GRU, and LSTM based NN architectures were constructed, followed by a fully connected layer. The models were hyper-tuned via Bayesian optimization across 20 trials. Learning rates ranged from $10^{-4}$ to $10^{-2}$, batch sizes were {32, 64, 128}, hidden units were {32, 64, 128, 192, 256}, and optimizers included Adam, Root Mean Square Propagation (RMSProp), and Stochastic Gradient Descent (SGD). Validation MSE results are shown in Tables 1 and 2. As can be seen from the results, GRU showed slightly lower MSE scores, which is also reflected in the FLC predictions shown in Figs. 9 and 11, where the LSTM predictions become worse than the GRU predictions as the number of increments increases.

Next, a specialized RNN layer was built following Eq. (18), substituting *Z* and *M* (Eqs. (21)–(22)) for *G* and *I*. This layer was followed by a fully connected sigmoid output. The architecture was trained over 8 trials, varying hidden units {64, 128, 192} and learning rate $[10^{-3},\,10^{-6}]$. The micro-step size *v* in Eq. (8) was fixed at $7\times 10^{-6}$, computed as the average incremental strain difference divided by 400 across the training set (Eq. (24)). Bayesian optimization guided the search, and each model was trained for up to 100 epochs. The validation results are shown in Table 3.

24 $$\begin{aligned} \begin{aligned} d\varepsilon _{1}^i&= \varepsilon _{1}^i - \varepsilon _{1}^{i-1}, \qquad d\varepsilon _{2}^i = \varepsilon _{2}^i - \varepsilon _{2}^{i-1}, \\ \delta \varepsilon _{i}&= \sqrt{(d\varepsilon _{1}^i)^2 + (d\varepsilon _{2}^i)^2}, \\ v&= \frac{1}{400N}\sum _{i=1}^{N}\sum _{j=1}^{400}\delta \varepsilon _{j}. \end{aligned} \end{aligned}$$

#### Discussion of the results

The bilinear FLCs with 100, 000-increment strain paths were used to evaluate consistency between the best-performing GRU and C-RNN models. Each path was downsampled to 400, 1000, and 10,000 increments using intervals of 250, 100, and 10, respectively. Localization points were estimated by both models for each discretization shown in Fig. 9.

As shown in Fig. 9A and B, for 400-increment paths, the GRU architecture achieves lower MSE than the specialized RNN for all three FLC cases, consistent with visual results in Fig. 9A. However, as the number of increments increases, the GRU predictions diverge, yielding higher MSE than the specialized RNN. The specialized RNN maintains nearly identical localization estimates across different discretizations (Fig. 9B), confirming **consistency**.

**Fig. 10:**
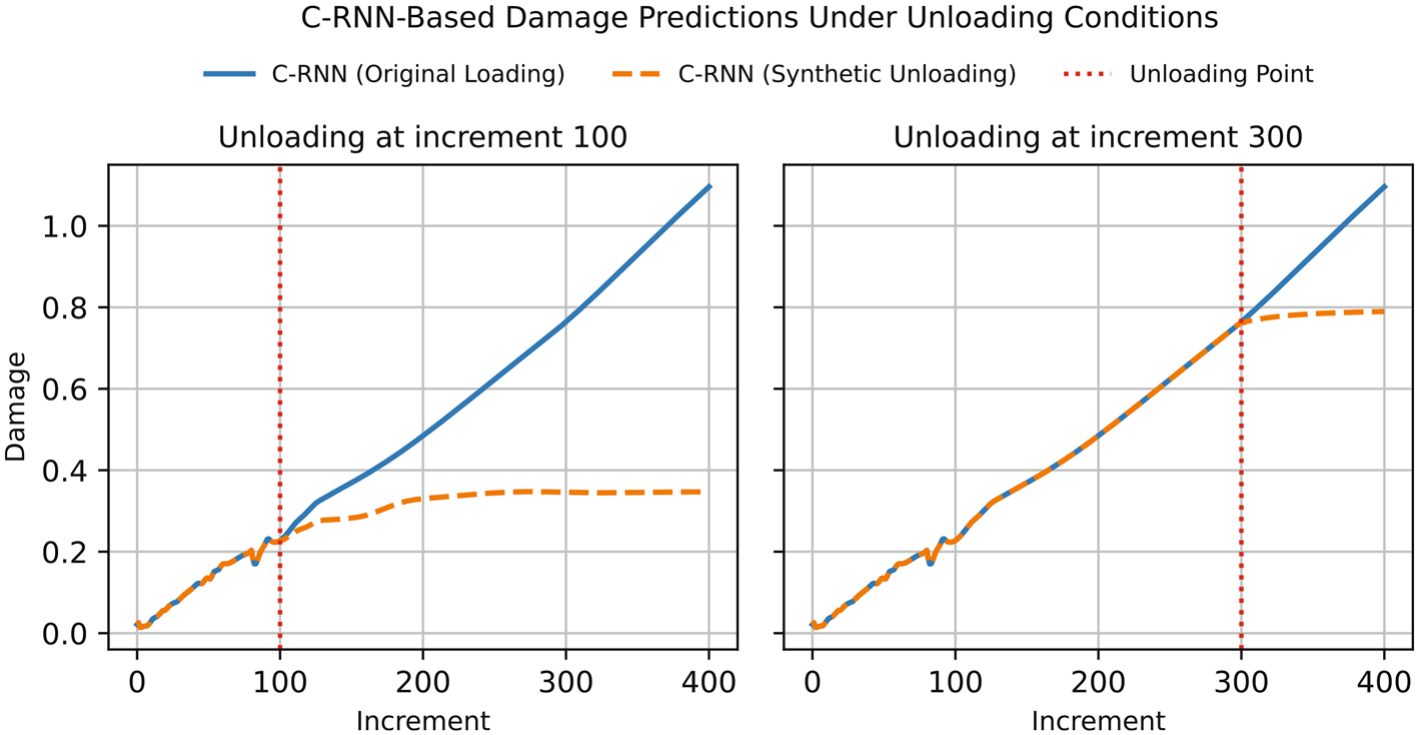
As can be seen from the two unloading scenarios, where unloading occurs at increments 100 and 300, the predicted damage continues to increase even after unloading. This behavior is physically inconsistent, as damage evolution is expected to remain constant under unloading conditions. However, the present study focuses specifically on monotonic and bilinear deformation paths. This limitation will be examined in future work, where the C-RNN formulation can be extended to account for unloading by enforcing zero damage updates during such conditions.

It should be noted that the present study focuses on monotonic and bilinear deformation paths. In unloading scenarios, where the incremental difference becomes zero and the same time-step strain values are provided, the proposed transition function may still update the hidden state, although no additional deformation accumulates. An empirical example is shown in Fig. 10, where the damage predictions still increase after the unloading points, which creates a physically inconsistent behavior. This behavior reflects a limitation of the current transition function formulation. An explicit rule for unloading scenarios could be embedded into the formulation in future work.

#### Computational complexity

The computational complexity of NN architectures plays a critical role in their practical applicability as surrogates in FE simulations. The experimental results on the bilinear loading dataset demonstrate that the proposed RNN-cell-based architecture provides consistent predictions, whereas the GRU-based model does not exhibit the same consistency. However, these architectures also differ significantly in computational cost during inference. Since inference speed is the dominant consideration in FE-coupled applications (while training cost is typically less critical), we evaluate and compare the computational complexity of the two models during inference.

The time complexity of a GRU-based NN architecture can be estimated from the update equations in Eq. (21), which define the general formulation of GRU layers. For the bilinear loading dataset, the input vector size is $|x_t|=3$. Denoting the hidden unit size by *h* and the input sequence length by *T*, the time complexity of the update functions *Z* and *R* in the GRU layer is $\mathcal {O}(T \cdot (3h + h^2))$. The element-wise operations in the gates $\hat{S}$ and *G* further increase the complexity to $\mathcal {O}(T \cdot (6h + h^2))$. Including the final output layer, the total time complexity for the GRU-based NN becomes:

$$\mathcal {O}(T \cdot (7h + h^2)).$$

For the customized RNN-based NN architecture, the total time complexity for the update functions *G* and *I* is equivalent to that of the GRU layer, i.e., $\mathcal {O}(T \cdot (6h + h^2))$. However, matrix multiplication in the final update function *F* (Eq. (19)) and in the output layer adds an additional term, yielding:

$$\mathcal {O}(T \cdot (7h + 2h^2)).$$

Although both architectures share the same asymptotic time complexity in Big-$\mathcal {O}$ notation, the customized RNN has a larger constant factor and coefficient overhead due to the additional operations in its transition function. Consequently, while the proposed architecture offers greater consistency in predictions, its computational cost grows more rapidly with the number of increments compared to the GRU-based model. This trade-off is an important consideration for deploying the customized RNN as a surrogate in large-scale FE simulations.

**Fig. 11:**
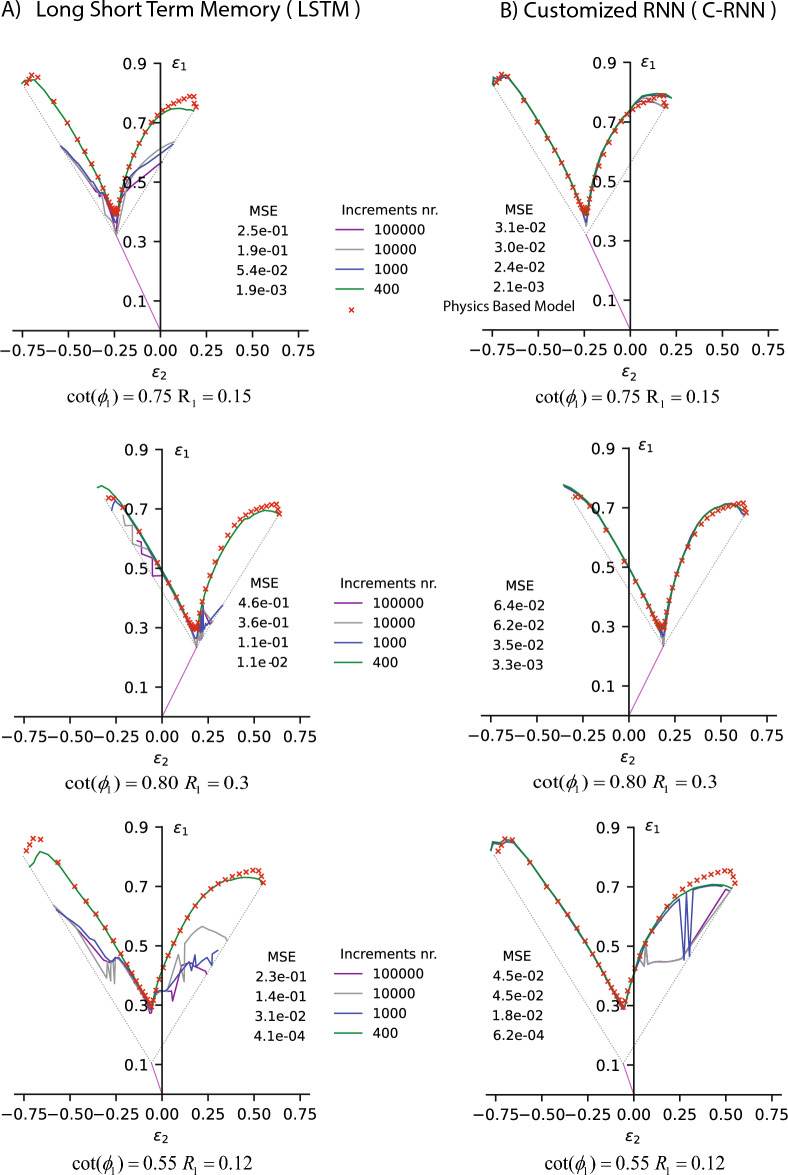
Bilinear Forming Limit Curve (FLC) estimates obtained from two NN architectures: (**A**) an LSTM-based model and (**B**) the proposed C-RNN. The LSTM predictions exhibit serious divergence as the number of increments increases, whereas the proposed RNN maintains consistent estimates across different strain-path discretizations. Additionally, as can be seen from the LSTM predictions, they perform worse than the GRU predictions, which also supports the MSE validation.

#### Future work

Several directions can be pursued to extend this study. First, the fixed step-size parameter *v*, currently predefined based on the simulation data, can be made *adaptive* by defining it as a learnable parameter within the architecture. By allowing *v* to evolve dynamically with the time step $x_{t}$, the model could learn the natural temporal scaling of deformation paths, improving both generalization and fidelity. This extension would not only broaden the concept of consistency but also enhance the physical interpretability and accuracy of damage predictions by coupling the adaptive step size with the underlying incremental deformation physics.

Additional future work includes testing the developed C-RNN-based surrogate model within a FEM-based abaqus environment. Currently, the NN architecture has been trained, developed, and tested on FE-based extracted data using Python and TensorFlow frameworks. The integration of this surrogate model into FEM-based abaqus simulations is under active development, where the proposed C-RNN architecture is embedded through a Fortran implementation as a constitutive update mechanism. In this setting, minor and major strain increments are used as inputs, and damage is directly predicted at each predefined increment step, while different increment frequencies are also considered to evaluate the model’s behavior within the simulation environment. This integration enables the assessment of the model’s performance under realistic structural simulation conditions, including both predictive accuracy and computational efficiency. Similar non-intrusive implementations of neural-network-based constitutive models, such as flow rule approximations for metallic materials, have been successfully integrated into abaqus user subroutines (e.g., VUHARD), demonstrating strong agreement with classical formulations and efficient computational performance^37,38^. These studies support the feasibility of embedding data-driven constitutive models into FE solvers. However, since this implementation involves additional data collection, numerical experiments, and extensive validation, it is currently being prepared as a separate research work and remains beyond the scope of the present study, where the primary objective is to develop a customized RNN formulation that satisfies the defined fundamental consistency requirements.

Third, although this study focused on bilinear deformation paths for training and validation, the proposed RNN architecture can be further evaluated on three-dimensional multiaxial loading paths to demonstrate the generalizability and robustness of the proposed consistency framework.

Fourth, the methodology could be extended beyond simulation-based datasets and validated directly on experimental measurements representing real material behavior under complex loading. Such validation would further strengthen the physical relevance of the proposed approach.

Fifth, the predictive accuracy of the customized RNN could be improved by incorporating gating mechanisms similar to those in GRU or LSTM architectures into the consistency-enforcing transition structure. This hybrid variant could be tested on FE-generated datasets to determine whether it preserves **consistency** while achieving higher predictive accuracy.

Finally, the proposed RNN formulation can be further extended to incorporate physically consistent rules for zero-state updates under unloading scenarios. This would require a reformulation of the transition function to better reflect the underlying structure of strain-path-dependent material behavior. Such an extension has the potential to generalize the framework beyond monotonic and bilinear loading conditions, enabling robust modeling of more complex and realistic deformation histories.

## Conclusion

For a NN architecture to function as a surrogate model within explicit FE simulations, its predictions must remain compatible with the fundamental physical properties of deformation histories and their resulting material damage. Accordingly, this study identifies and formalizes two key requirements: **truncation**, and **consistency**. The first, **truncation**, requires that inputs from future time steps do not influence damage predictions at prior time steps. The second, **consistency**, arises from the incremental nature of FE simulations where the same deformation path may be discretized into different numbers of increments without altering the underlying physical response. Thus, a consistent surrogate model must produce identical damage predictions regardless of the chosen increment size.

Inspired by Bonatti and Mohr’s work on **consistency** in constitutive modeling^26^, a specialized RNN transition formula was proposed to address these two requirements. The new formulation enforces consistency by operating directly on time-step strain inputs rather than incremental differences. Two one-layer NN architectures were implemented: one based on a standard GRU and another employing the proposed C-RNN cell, each followed by a fully connected output layer. Both were trained and hyper-tuned using Bayesian optimization on 400-increment bilinear deformation paths, varying learning rates, hidden sizes, batch sizes, and optimizer types.

The results demonstrated that while the GRU-based model achieved slightly higher accuracy for low discretizations, its predictions diverged with increasing increments. In contrast, the specialized RNN maintained stable and physically consistent estimates across all tested discretizations. This confirms the theoretical hypothesis that embedding **truncation**, and **consistency** within the RNN transition dynamics enables physically grounded, discretization-independent predictions.

The proposed framework provides a structured approach for developing NN architectures that respect the intrinsic physical constraints of deformation history learning. It offers a pathway toward integrating data-driven surrogate models into computational mechanics workflows, enabling reliable real-time prediction of material damage and localization phenomena within FE environments.

The best-performing architectures from both models were used to predict 1,000, 10,000, and 100,000 increments to estimate bilinear FLCs. The results demonstrate that the C-RNN based NN architecture satisfies both fundamental requirements **truncation** and **consistency** while accurately estimating damage states across varying discretizations. Furthermore, the proposed RNN cell exhibits potential for adaptability across different deformation scenarios. To support this observation, additional experiments were conducted on a synthetically generated dataset containing strain paths with no physically derived dependence, suggesting that the proposed transition formulation can operate beyond physically constrained data. This unique RNN transition mechanism may serve as a basis that can be further extended and refined in future research, supporting continued development of data-driven surrogate modelling approaches.

Another important aspect of introducing the **truncation** and **consistency** requirements lies in emphasizing the balance between rapid advancements in artificial intelligence and the fundamental principles of physics. While increasingly complex NN architectures are being developed and often achieve high predictive accuracy, the underlying physics-based rules and constraints are frequently overlooked or receive limited attention. These fundamental requirements, however, remain essential when designing NN models for physical systems. Since NN perform purely mathematical mappings based on the data provided, which often originates from physics-based simulations, it is important to incorporate intrinsic physical rules to prevent non-physical or misleading outcomes. In this study, the proposed formulation demonstrates how physical consistency requirements can be embedded within an NN framework, which may support further research on integrating physics-based constraints into data-driven models.

## Data Availability

Data and research code supporting this study are available at https://zenodo.org/records/11914409
